# Platelet Parameters and Their Relationships With the Thickness of the Retinal Nerve Fiber Layer and Ganglion Cell Complex in Primary Open-Angle Glaucoma

**DOI:** 10.3389/fneur.2022.867465

**Published:** 2022-05-02

**Authors:** Yi Ma, Shengjie Li, Mingxi Shao, Wenjun Cao, Xinghuai Sun

**Affiliations:** ^1^Department of Clinical Laboratory, Shanghai Medical College, Eye and Ear Nose and Throat Hospital, Fudan University, Shanghai, China; ^2^Department of Ophthalmology and Visual Science, Shanghai Medical College, Eye and Ear Nose and Throat Hospital, Fudan University, Shanghai, China; ^3^State Key Laboratory of Medical Neurobiology, Institutes of Brain Science and Collaborative Innovation Center for Brain Science, Fudan University, Shanghai, China; ^4^National Health Commission Key Laboratory of Myopia (Fudan University), Shanghai, China; ^5^Key Laboratory of Myopia, Chinese Academy of Medical Sciences, Shanghai, China; ^6^Shanghai Key Laboratory of Visual Impairment and Restoration (Fudan University), Shanghai, China

**Keywords:** primary open-angle glaucoma, platelet, retinal nerve fiber layer, ganglion cell complex, optical coherence tomography

## Abstract

**Objective:**

Glaucoma is a neurodegenerative disease of the visual system. Platelet parameters are correlated with visual field mean deviation (MD) in glaucoma, but there is a lack of relative data on their relationship with structural changes in the retina. This study aimed to explore the relationship between platelet parameters and retinal nerve fiber layer (RNEL), ganglion cell complex (GCC) thickness, and cup/disk area ratio, evaluated by optical coherence tomography (OCT) in primary open-angle glaucoma (POAG).

**Methods:**

A total of 118 consecutive patients with POAG and 120 age- and sex-matched control subjects were included in this retrospective study. Demographic data, platelet parameters in blood tests, visual field, and OCT results were evaluated. The RNFL was divided into the temporal, superior, nasal, and inferior quadrants. Based on the visual field MD, the patients were stratified into mild (MD ≤ 6.0 dB), moderate (6 dB < MD ≤ 12 dB), and severe (MD > 12.0 dB) subgroups.

**Results:**

Patients with POAG had significantly lower platelet (PLT) levels and significantly higher platelet distribution width (PDW) and mean platelet volume (MPV) levels than controls. As the visual field MD increased, structural evaluation by OCT identified loss of disk rim area, average GCC thickness, and average RNFL thickness (all *P* < 0.001), as well as increased PDW (*P* < 0.001) and MPV (*P* = 0.004) levels in patients with POAG. The Spearman's rank correlation analysis showed that PDW levels were significantly correlated with OCT parameters such as RNFL thickness (*r* = −0.370, *P* < 0.001), GCC thickness (*r* = −0.294, *P* = 0.001), and cup/disk area ratio (*r* = 0.322, *P* < 0.001), as well as visual field MD (*r* = 0.607, *P* < 0.001) and mean sensitivity (MS) (*r* = −0.570, *P* < 0.001). Significantly correlations were also found between MPV and RNFL thickness (*r* = −0.321, *P* < 0.001), GCC thickness (*r* = −0.194, *P* = 0.041), and cup/disk area ratio (*r* = 0.237, *P* = 0.010). All the quadrants showed similar negative correlations between PDW, MPV, and RNFL thickness. The multiple linear regression analyses showed significant association between PDW and RNFL thickness (β = −0.331, *P* < 0.001), PDW and GCC thickness (β = −0.288, *P* = 0.002), MPV and RNFL thickness (β = −0.313, *P* = 0.001), and MPV and GCC thickness (β = −0.188, *P* = 0.048).

**Conclusion:**

This study found significantly negative association between PDW, MPV levels and RNFL, GCC thickness, as well as positive association between PDW, MPV levels, and cup/disk area ratio in patients with POAG, suggesting that platelet activation may contribute to glaucomatous optic neuropathy.

## Introduction

Glaucoma is a neurodegenerative disease of the visual system and leading cause of irreversible blindness worldwide (1). Primary open-angle glaucoma (POAG), as the most prevalent form of glaucoma, is described as a multifactorial optic neuropathy that is chronic and progressive with a characteristic acquired loss of optic nerve fibers. In addition to increased intraocular pressure, many factors are involved in optic nerve damage, such as insufficient blood supply to the eye, vascular dysfunction, and breakdown of neurovascular coupling in glaucoma. Despite intense study, the pathogenesis of POAG is still not completely understood. There is also an urgent need for clinical discovery of new biomarkers and critical targets for early diagnosis and early treatment.

Platelets are anucleate cells that circulate in blood and are critical for the body's response to vascular injury, as well as preventing bleeding; emerging evidence suggests that platelet plays essential functions in several neurodegenerative conditions (2), including glaucoma, Parkinson's disease (PD), and Alzheimer's disease (AD). Platelet has been demonstrated to be involved in the pathomechanisms of glaucoma through animal model and clinical evidence (3–5). Williams et al. (3) confirmed the monocyte-platelet interactions in the optic nerve head and proposed targeting platelet aggregation and activation in glaucoma treatment. Nishijima et al. (4) observed that platelets actively interacted with retinal endothelial cells in the post-ischemic retina through P-selectin. Some observed an increased platelet aggregation in glaucoma, which negatively influences blood flow in the small branches of the short ciliary arteries supplying the optic disk (5). It is also vital to note that platelets show high expression of several proteins associated with the development of glaucoma and AD, such as the amyloid precursor protein (APP) and tau protein (6). Together this, it makes it interesting to consider the contribution of platelets to the pathomechanism of neurodegeneration.

Primary open-angle glaucoma is characterized by progressive loss of retinal ganglion cells and typical glaucomatous visual field defects. Retinal nerve fiber layer (RNFL) around the optic nerve head and in the macular region can be reproducible quantitative measured by optical coherence tomography (OCT). Our previous study reported that mean platelet distribution width (PDW) and mean platelet volume (MPV), two of which are platelet parameters implying platelet function and activation, were significantly elevated in patients with POAG and were positively associated with visual field mean deviation (MD) (7). However, whether platelet parameters are associated with retinal thickness, which represent structural change in glaucoma, is still unknown. We performed the following retrospective study to explore the possible correlation between platelet parameters and RNFL, ganglion cell complex (GCC) thickness, and cup/disk area ratio, evaluated by OCT in POAG.

## Methods

This study was approved by the Ethics Review Committee of Eye, Ear, Nose, and Throat (EENT) Hospital, Fudan University. The design and implementation of this study adhered to the tenets of the Declaration of Helsinki. A written informed consent was obtained from all the patients.

### Subjects

#### Patients With Primary Open-Angle Glaucoma

Patients with POAG were consecutively enrolled in the Eye and ENT Hospital of Fudan University between January 2017 and December 2019. The definition of POAG was based on: (1) glaucomatous optic neuropathy such as a vertical cup disk ratio (VCDR) > 0.7 or an intereye asymmetry of > 0.2, with notching, rim thinning, or retinal nerve fiber layer (RNFL) defect; (2) typical visual field defect that corresponds to the structural change: presence of at least three contiguous non-edged test points within the same hemifield on the corrected probability plot at *P* < 0.05, with at least one point *P* < 0.01, excluding points directly above and below the blind spot; (3) elevated intraocular pressure (IOP) (>21 mm Hg); and (4) the anterior chamber angle was considered open and normal in appearance on gonioscopy for both the eyes (8). Most of the patients with POAG received topical glaucoma medications. Patients with POAG were divided into the three groups based on the progression of their optic nerve damage as measured by visual field: mild (MD ≤ 6.0 dB), moderate (6 dB < MD ≤ 12 dB), and severe (MD > 12.0 dB) (9).

**Figure 1:**
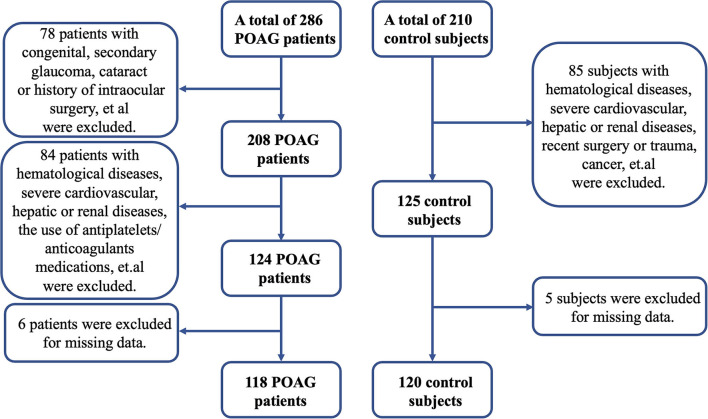
The flowchart of the study regarding subject recruitment.

Excluded from this study were patients with congenital, secondary glaucoma, or a history of intraocular surgery. Patients with concomitant ocular diseases, which could potentially impair visual fields such as optic disk anomalies, optic nerve diseases, retinal diseases, pathologic myopia, and intracranial lesions, were excluded. A review of systemic diseases was conducted; patients met the following criteria were also excluded: (1) <18 years old or pregnant woman; (2) hematological diseases such as aplastic anemia, purpura hemorrhagica, and primary thrombocytosis; (3) abnormal coagulation function; (4) severe cardiovascular, hepatic, or renal diseases; (5) recent surgery or trauma; (6) cancer; (7) acute infectious diseases or autoimmune diseases; (8) thyroid dysfunction; and (9) the use of antiplatelets/anticoagulants medications during the previous 6 months such as aspirin, clopidogrel, warfarin, and cilostazol (7). Initially, 286 patients were recruited. Based on the inclusion and exclusion criteria, 168 patients were excluded. Figure 1 depicts the flowchart of the study regarding patient recruitment.

#### Healthy Controls

Healthy controls were consecutively recruited from individuals who participated in yearly health screenings during this study period. The inclusion criteria were as follows: IOP <21 mm Hg; age 18 years and older; normal-appearing optic disks; anterior chamber angle open; and VCDR ≤ 0.5. The exclusion criteria were as follows: family history or personal history of glaucoma; complaints of eye discomfort; prior ocular trauma or surgery; severe systemic diseases; and the use of antiplatelets/anticoagulants medications during the previous 6 months. According to the inclusion and exclusion criteria, as shown in Figure 1, a total of 120 control subjects were finally included in this study.

### Clinical Examinations

All the enrolled POAG subjects in this study were inpatients and blood samples were drawn for laboratory measurements on the same day that standardized ophthalmic examination and comprehensive physical examination were performed.

#### Ophthalmic Examination

All the subjects underwent complete ophthalmological examination conducted by a glaucoma specialist, which included slit-lamp-assisted biomicroscope, gonioscopy (Haag-Streit, Bern, Switzerland), a three-time IOP measurement using Goldmann applanation tonometry (Haag-Streit, Bern, Switzerland), and then averaged the analysis of the eye's fundus using a digital retinal camera (TRC-NW200, Topcon), visual field examinations, and spectral-domain optical coherence tomography (SD-OCT) tests. Visual fields mean deviation (MD) and visual fields mean sensitivity (MS) were measured with the Octopus automated perimeter (Haag-Streit, Bern, Switzerland).

All the patients had a minimum of three visual field tests. Considering the learning effect of the visual field tests, the results of the first two tests were excluded. Only reliable (a false positive/negative below 15% and a reliability factor below 20%) and compatible visual field results were included. Each patient had a minimum of one reliable visual field test (10). Each control individual underwent preliminary ophthalmic examinations, which included refractive status, gonioscopy, slit-lamp biomicroscopic examination, and IOP, as carried out by glaucoma specialists.

Spectral-domain optical coherence tomography was performed using the RTVue-100 system (Optovue Incorporation, Fremont, California, USA) to measure the RNFL thickness, GCC thickness, and cup/disk area ratio. The RNFL thickness was measured automatically at a 3.45-mm diameter around the center of the optic disk, using the optic nerve head (ONH) scanning protocol software. The RNFL thickness parameter was designed to evaluate the mean thickness in 360° area. The RNFL was divided into temporal (316–45°), superior (46–135°), nasal (136–225°), and inferior (226–315°) quadrants. In this study, the GCC was defined as the distance between the internal limiting membrane to the inner plexiform layer. The GCC scanning protocol was used for measuring the GCC thickness. The GCC within the central 6 mm diameter area of the macular was calculated. Measurements were obtained by a well-trained operator. Criteria for acceptable OCT images were a signal strength index (SSI) >35, no marked eye movements during the examination, and no black bands caused by blinking during the examination.

#### Clinical Information and Laboratory Measurements

Clinical and demographic information was obtained from the medical data platform of the hospital. Medical examinations, including the assessment of ECG, X-ray, liver function, infectious diseases, renal function, blood pressure, heart rate, visual acuity, body temperature, and body mass index (BMI), were performed for all the subjects at the Eye and ENT Hospital of Fudan University. The BMI was calculated as weight in kilograms divided by height in meters squared.

Laboratory measurements were performed in the department of clinical laboratory science of the Eye and ENT Hospital. Blood samples for platelet parameters, including platelet (PLT) count, PDW, and MPV, were taken in laboratory tubes with ethylenediaminetetraacetic acid (EDTA) and analyzed by an automated hematology analyzer (Sysmex XN 1000, Japan) within 30 min following standard venipuncture of the veins in the antecubital fossae (anterior elbow veins). The reference range of PLT, MPV, and PDW was 100–400 × 10^9^/L, 9.0–16.0, and 9.0–17.0 fL, respectively. Quality control of the automated hematology analyzer was performed each day before sample detection with three levels (low, medium, and high) of quality control materials. Internal laboratory quality controls were analyzed daily over the 3-year period, with no significant changes in the values.

### Statistical Analyses

All the analyses were performed using SPSS version 23.0 (IBM SPSS Statistics) and Microsoft Excel 2016. The figures were created by GraphPad Prism version 7 software (California, USA). Data are presented as mean ± SD or medians (interquartile ranges) according to data distribution. Normality was assessed using the Kolmogorov–Smirnov test. The independent Student's *t*-test and chi-squared test were used for the comparison of participant characteristics between the POAG group and the control group. Differences of ocular and platelet parameters among the 3 disease-severity groups were tested with the Kruskal–Wallis test or 1-way ANOVA. Correlations between platelet parameters and ocular parameters in POAG were analyzed using the Spearman's rank correlation analysis. Additionally, patients with POAG were divided into 2 subgroups according to mean PDW (PDW > 13.69 vs. <13.69 fL group) and mean MPV levels (MPV > 10.44 vs. <10.44 fL group) to further evaluate the differences of their OCT parameters. Finally, the multivariate linear regression analyses, adjusted for age, gender, body mass index, and intraocular pressure, were performed to evaluate the association between PDW, MPV, and OCT parameters. A two-sided *P* < 0.05 was considered as statistically significant.

## Results

### Subject Characteristics

According to the inclusion and exclusion criteria, a total of 118 patients with POAG and 120 control subjects were enrolled. If both the eyes of the same individual were affected by POAG, only one eye was randomly selected. Table 1 summarizes the demographic and clinical characteristics of the participants. The POAG group and the control group were closely matched in terms of mean age (*P* = 0.551), gender (*P* = 0.783), and BMI (*P* = 0.806). Compared with the control subjects, patients with POAG had significantly lower PLT levels (*P* = 0.037) and significantly higher PDW (*P* < 0.001) and MPV (*P* = 0.038) levels. Table 1 also shows ocular parameters, including IOP, visual field, and OCT parameters, of patients with POAG.

**Table 1:** Characteristics of the study subjects.

	**POAG (*n* = 118)**	**Control (*n* = 120)**	***P*-Value^a^**
Age (y)	51.52 ± 14.63	50.53 ± 9.80	0.551
Female/Male	37/81	40/80	0.783
BMI	23.93 ± 4.11	24.06 ± 3.98	0.806
PLT (10^9^/L)	206.65 ± 52.77	220.52 ± 48.83	**0.037**
Male	201.66 ± 51.16	216.73 ± 47.43	**0.040**
Female	216.76 ± 54.02	240.30 ± 50.43	**0.022**
PDW (fL)	13.69 ± 3.29	11.82 ± 2.69	**<0.001**
Male	13.60 ± 3.35	11.76 ± 2.53	**<0.001**
Female	14.53 ± 2.87	11.89 ± 3.02	**<0.001**
MPV (fL)	10.44 ± 1.29	10.10 ± 1.16	**0.038**
Male	10.32 ± 1.37	10.07 ± 1.11	**0.041**
Female	10.85 ± 1.02	10.19 ± 1.29	**0.026**
IOP (mm Hg)	20.00 (15.98–29.70)	–	–
MD (dB)	15.20 (8.57–24.05)	–	–
MS (dB)	14.40 (5.90–20.80)	–	–
RNFL thickness (μm)	68.22 (59.00–80.00)	–	–
GCC thickness (μm)	69.42 (61.18–79.83)	–	–
Cup/Disc area ratio	0.76 (0.65–0.85)	–	–

*Data distribution was tested using the Kolmogorov Smirnov test. Data are expressed as mean ± SD or medians (interquartile ranges) according to the data distribution.*

a *Differences between groups were tested with the independent-samples T-test and β^2^ test.*

*POAG, primary open-angle glaucoma; BMI, body mass index; PLT, platelet count; PDW, platelet distribution width; MPV, mean platelet volume; IOP, intraocular pressure; MD, visual field mean deviation; MS, visual field mean sensitivity; RNFL, retinal nerve fiber layer; GCC, ganglion cell complex. Bold values indicates P < 0.05*.

### Association Between Platelet Parameters and Optical Coherence Tomography Parameters in Primary Open-Angle Glaucoma

Table 2 presents an overview of the ocular parameters and platelet parameters in patients with POAG, stratified according to severity. As the severity of the disease increased, structural evaluation by OCT identified loss of average GCC thickness and average RNFL thickness (all *P* < 0.001), in addition to increased cup/disk area ratio (*P* < 0.001). As for the platelet parameters, the PDW (*P* < 0.001) and MPV (*P* = 0.004) levels were highest in the severe POAG group, followed by the moderate POAG group and then the mild POAG group.

**Table 2:** Ocular parameters and platelet parameters in patients with POAG, stratified according to severity.

	**Mild POAG (*n* = 20)**	**Moderate POAG (*n* = 30)**	**Severe POAG (*n* = 68)**	***P*-Value^a^**
IOP (mmHg)	18.00 (15.00–22.30)	19.30 (14.83–22.35)	22.00 (17.00–32.35)	0.012
**Visual field**
MD (dB)	4.55 (2.20–5.15)	9.75 (8.26–10.73)	21.35 (16.95–26.58)	**<0.001**
MS (dB)	23.70 (22.25–25.68)	17.60 (16.80–20.20)	7.10 (2.70–10.95)	**<0.001**
**Structural assessments**
RNFL thickness (μm)	83.00 (77.69–90.19)	75.50 (67.50–89.63)	60.72 (56.91–68.59)	**<0.001**
GCC thickness (μm)	85.98 (79.71–95.68)	75.00 (68.37–86.57)	62.00 (56.74–70.22)	**<0.001**
Cup/Disc area ratio	0.58 (0.51–0.70)	0.69 (0.64–0.80)	0.73 (0.73–0.86)	**<0.001**
**Platelet parameters**
PLT (10^9^/L)	213.88 ± 44.57	209.17 ± 51.63	203.47 ± 47.44	0.697
PDW (fL)	11.14 ± 2.02	12.33 ± 3.19	15.04 ± 2.91	**<0.001**
MPV (fL)	9.84 ± 0.74	9.93 ± 1.24	10.71 ± 1.44	**0.004**

*Data distribution was tested using the Kolmogorov Smirnov test. Data are expressed as mean ± SD or medians (interquartile ranges) according to the data distribution.*

a *Differences between groups were tested with the Kruscal-Wallis test or 1-way analysis of variance (ANOVA).*

*POAG, primary open-angle glaucoma; IOP, intraocular pressure; MD, visual field mean deviation; MS, visual field mean sensitivity; RNFL, retinal nerve fiber layer; GCC, ganglion cell complex; PLT, platelet count; PDW, platelet distribution width; MPV, mean platelet volume. Bold values indicates P < 0.05*.

**Figure 2:**
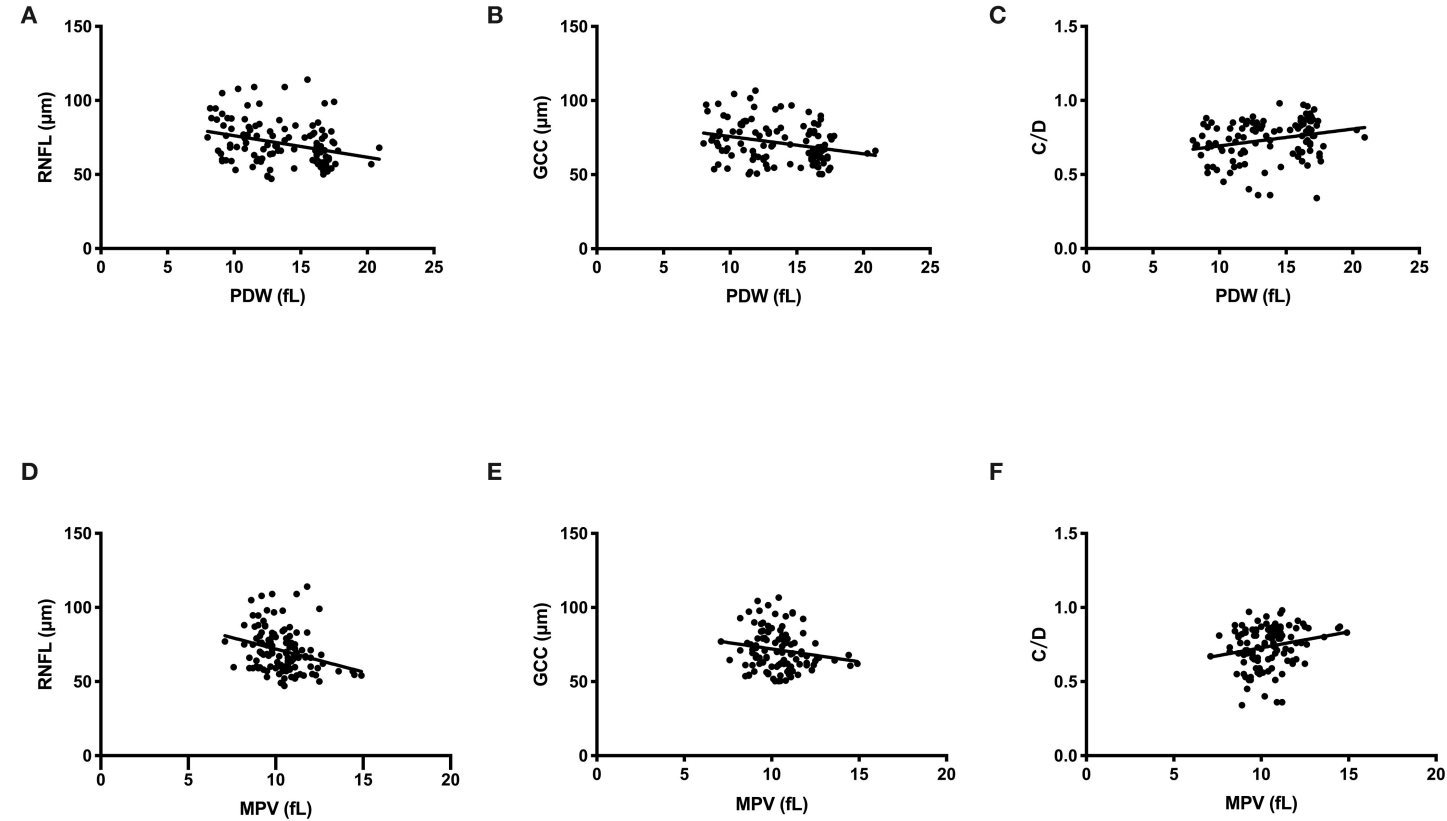
Scatterplot of patient individual measurements for platelet parameters and optical coherence tomography (OCT) parameters. Each data point represents one patient. Linear regression is displayed. **(A)** PDW and RNFL thickness. **(B)** PDW and GCC thickness. **(C)** PDW and C/D. **(D)** MPV and RNFL thickness. **(E)** MPV and GCC thickness. **(F)** MPV and C/D. PDW, platelet distribution width; MPV, mean platelet volume; RNFL, retinal nerve fiber layer; GCC, ganglion cell complex; C/D, cup/disc area ratio.

The Spearman's rank correlation analysis showed that the PDW levels were significantly correlated with visual field MD (*r* = 0.607, *P* < 0.001), MS (*r* = −0.570, *P* < 0.001), OCT parameters average RNFL thickness (*r* = −0.370, *P* < 0.001, Figure 2A), average GCC thickness (*r* = −0.294, *P* = 0.001, Figure 2B), and cup/disk area ratio (*r* = 0.322, *P* < 0.001, Figure 2C). Significant correlations were also found between MPV levels and visual field MD (*r* = 0.291, *P* = 0.001), MS (*r* = −0.336, *P* = 0.001), OCT parameters average RNFL thickness (*r* = −0.321, *P* < 0.001, Figure 2D), average GCC thickness (*r* = −0.194, *P* = 0.041, Figure 2E), cup/disk area ratio (*r* = 0.237, *P* = 0.010, Figure 2F), as shown in Table 3 and Figure 2. In addition, the association between visual field parameters and retinal thickness was also confirmed. RNFL (*r* = −0.658, *P* < 0.001) and GCC (*r* = −0.639, *P* < 0.001) thicknesses were negatively associated with MD; moreover, RNFL (*r* = 0.717, *P* < 0.001) and GCC (*r* = 0.704, *P* < 0.001) thicknesses were positively associated with MS.

**Table 3:** Correlation between platelet parameters and ocular parameters in POAG.

	**PLT**	**PDW**	**MPV**
MD	*r* = −0.087	*r* = 0.607	*r* = 0.291
	*P* = 0.348	***P*** **<** **0.001**	***P*** **=** **0.001**
MS	*r* = 0.157	*r* = −0.570	*r* = −0.336
	*P* = 0.128	***P*** **<** **0.001**	***P*** **=** **0.001**
RNFL	*r* = 0.151	*r* = −0.370	*r* = −0.321
	*P* = 0.105	***P*** **<** **0.001**	***P*** ** <0.001**
GCC	*r* = 0.121	*r* = −0.294	*r* = −0.194
	*P* = 0.200	***P*** **=** **0.001**	***P*** **=** **0.041**
C/D	*r* = −0.097	*r* = 0.322	*r* = 0.237
	*P* = 0.294	***P*** **<** **0.001**	***P*** **=** **0.010**

*Spearman correlation was used. A P <0.05 was considered statistically significant.*

*POAG, primary open-angle glaucoma; MD, visual field mean deviation; MS, visual field mean sensitivity; RNFL, retinal nerve fiber layer; GCC, ganglion cell complex; C/D, cup/disc area ratio; PLT, platelet count; PDW, platelet distribution width; MPV, mean platelet volume. Bold values indicates P <0.05*.

### Subgroup Analyses

Further, we explored the association between RNFL thickness in different quadrants and changes in platelet parameters. Temporal, superior, nasal, and inferior quadrants of RNFL thickness measurements of the cases were analyzed in POAG. Statistically significant correlations were found between PDW, MPV, and RNFL thickness in all the quadrants. The detailed information is shown in Table 4.

**Table 4:** Correlation between platelet parameters and RNFL thickness measurements by quadrants in POAG.

	**Temporal**	**Superior**	**Nasal**	**Inferior**
PDW	*r* = −0.262	*r* = −0.307	*r* = −0.286	*r* = −0.230
	*p* = 0.004	*p* = 0.001	*p* = 0.002	*p* = 0.012
MPV	*r* = −0.235	*r* = −0.343	*r* = −0.289	*r* = −0.219
	*p* = 0.010	*p* < 0.001	*p* = 0.002	*p* = 0.017

*Spearman correlation was used.*

*POAG, primary open-angle glaucoma; RNFL, retinal nerve fiber layer; PDW, platelet distribution width; MPV, mean platelet volume*.

**Figure 3:**
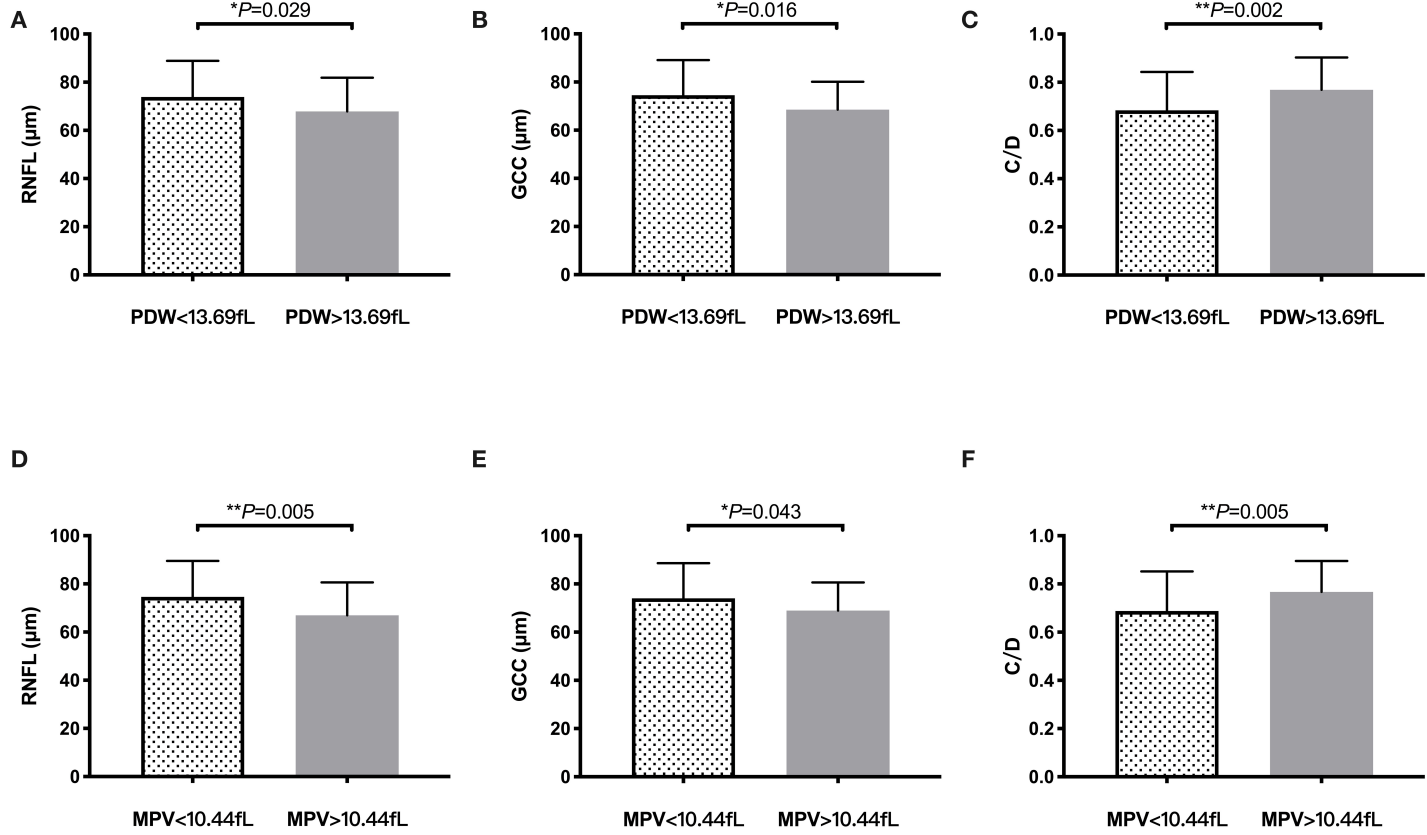
OCT findings according to PDW and MPV levels in primary open-angle glaucoma (POAG). Bar columns represent mean ± SD of OCT parameters in patients with POAG with different platelet PDW (PDW > 13.69 vs. <13.69 fL) and MPV (MPV > 10.44 vs. <10.44 fL) levels. *P < 0.05, **P < 0.01. **(A)** RNFL thickness in PDW subgroup. **(B)** GCC thickness in PDW subgroup. **(C)** C/D in PDW subgroup. **(D)** RNFL thickness in MPV subgroup. **(E)** GCC thickness in MPV subgroup. **(F)** C/D in MPV subgroup. PDW, platelet distribution width; MPV, mean platelet volume; RNFL, retinal nerve fiber layer; GCC, ganglion cell complex; C/D, cup/disc area ratio.

Additionally, patients with POAG were divided into 2 subgroups according to mean PDW and mean MPV levels to further evaluate the differences of their OCT parameters (Figure 3). Significantly thinner average RNFL thickness (*P* = 0.029, Figure 3A) and average GCC thickness (*P* = 0.016, Figure 3B), in addition with larger cup/disk area ratio (*P* = 0.002, Figure 3C), were found in patients with POAG with PDW > 13.69 fL than those of the other group. Similarly, patients with MPV > 10.44 fL also had significantly thinner average RNFL thickness (*P* = 0.005, Figure 3D), average GCC thickness (*P* = 0.043, Figure 3E), and significantly larger cup/disk area ratio (*P* = 0.005, Figure 3F) than those of patients with MPV <10.44 fL.

### Multiple Linear Regression Analyses

As shown in Table 5, the multiple linear regression analyses were performed to further analyze the association between PDW, MPV, and OCT parameters, with adjustment for age, gender, body mass index, and intraocular pressure. There was a statistically significant association between PDW and RNFL thickness (β = −0.331, *P* < 0.001), PDW and GCC thickness (β = −0.288, *P* = 0.002), MPV and RNFL thickness (β = −0.313, *P* = 0.001), and MPV and GCC thickness (β = −0.188, *P* = 0.048).

**Table 5:** Multiple linear regression analysis of platelet parameters and optical coherence tomography (OCT) parameters in POAG.

	**PDW β (*P*, 95% CI)**	**MPV β (*P*, 95% CI)**
RNFL	−0.331 (** <0.001**, −2.307 to −0.679)	−0.313 (**0.001**, −5.306 to −1.399)
GCC	−0.288 (**0.002**, −1.973 to −0.445)	−0.188 (**0.048**, −3.699 to −0.016)

*Multiple linear regression analyses were performed with adjustment for age, gender, body mass index and intraocular pressure.*

*95% CI, 95% confidence interval; POAG, primary open-angle glaucoma; PDW, platelet distribution width; MPV, mean platelet volume; RNFL, retinal nerve fiber layer; GCC, ganglion cell complex. Bold values highlight the P value of <0.05*.

## Discussion

Previously, we explored the relationship between platelet parameters and functional changes evaluated by visual field defects in POAG (7). In this study, further study on platelet and structural loss of retinal thickness found close correlations between PDW, MPV RNFL, and GCC thickness. In view of the fact that MPV or PDW reflects changes in platelet morphological and functional status, our findings provide clinical evidence to prove that platelet activation may contribute to glaucomatous optic neuropathy in POAG.

Platelet has been implicated in the pathogenesis of glaucoma through animal model and clinical evidence. Animal models have demonstrated that monocyte-platelet aggregates into the optic nerve head (ONH) are involved in early glaucomatous damage and that inhibition of platelet adhesion to the vessel wall can provide neuroprotection in glaucoma (3). Nishijima et al. (4) observed that platelets interacted actively with retinal endothelial cells in the post-ischemic retina through P-selectin expressed on the retinal endothelial cells. Several clinical studies found increased platelet aggregation (5, 11) and elevated PDW and MPV levels in POAG (7), indicating a prothrombotic state. Maric et al. (12) found higher MPV levels and nailfold capillary morphological vascular changes in patients with exfoliative glaucoma. Antiplatelet effect of antiglaucomatous eye drops was reported (13). In a word, altered platelet aggregability may trigger microthrombosis of the retinal capillaries and the short ciliary arteries, which result in vascular damage and generate defects in the microcirculation of the optic nerve head.

This study, using SD-OCT, an effective and reliable tool for monitoring structural changes of the retina, provided the information that the morphology and function of the platelet may be involved in the loss of retinal ganglion cells (RGCs). The evaluation of retinal thickness is as important as visual field testing for the evaluation of patients with glaucoma. It has been reported that RNFL loss of about 40 to 60% can occur before an appearance of visual field defect (14). In this study, structural assessments by OCT identified significant loss of disk rim area, average GCC thickness, and average RNFL thickness, in addition to elevated PDW and MPV levels, as the severity of POAG increased. Moreover, the Spearman's rank correlation analyses revealed significantly negative correlations between PDW, MPV levels, RNFL, and GCC thickness, in addition to positive correlations between PDW, MPV levels, and cup/disk area ratio.

Mean platelet volume and PDW are widely used for measuring the function and activation of platelet in the blood (15). To be specific, MPV is a marker of average platelet size and PDW represents the heterogeneity of platelet size. Increased MPV and PDW levels indicate more active and larger platelets that release more thromboxane A2 and aggregate more readily *in vitro* (16). In this study, significantly higher MPV and PDW levels and their correlation with visual field loss were found in POAG, which is consistent with our previous study (7). More importantly, we investigate the association between structural changes and platelet parameters. We found that PDW and MPV levels increased significantly as the RNFL and GCC thickness getting thinner and cup/disk area ratio getting larger, evaluated by OCT. To further explore the association between platelet parameters and retinal thickness, temporal, superior, nasal, and inferior quadrants of RNFL thickness were analyzed. All the four quadrants of RNFL thickness were found to be negatively associated with PDW and MPV, with the superior quadrant showing the strongest correlations with platelet parameters. These results demonstrated the agreement between platelet parameters and structural (OCT) and functional (VF) glaucomatous damage in POAG.

Figure 3 shows it directly that patients with POAG with higher PDW and MPV levels had significantly thinner RNFL and GCC thickness, as well as enlarged cup/disk area ratio, which suggested that platelet activation may contribute to the loss of RGCs and change of ONH in glaucoma. Several studies have reported the role of platelet in neurodegenerative disorders, such as Alzheimer's disease, Parkinson's disease, multiple sclerosis, and Huntington's disease (17). Platelets have the ability to rapidly adhere to the endothelium cells, become activated and secrete bioactive mediators, thus contributing to blood–brain barrier permeability, allowing the entry of white blood cells and producing cerebrovascular inflammation, which plays significant roles in neurons loss in neurodegeneration (18).

As age is known to be associated with RNFL loss (19), this was overcome by considering age as a covariate in our linear regression model. The multiple linear regression analysis further confirmed that RNFL and GCC thicknesses were independently associated with platelet parameters such as PDW and MPV (*P* < 0.05) in patients with POAG after adjusting for age, gender, body mass index, and intraocular pressure. Thus, measurement of platelet parameters may be a useful tool to predict structural damage in POAG, which is expected to be a simple, convenient, and efficient approach to supplement ophthalmic examination in glaucoma. Moreover, considering that platelet activation appears to be important to glaucomatous damage, targeting the regulation of platelet function may offer novel therapeutic interventions in glaucoma, especially in POAG.

It is increasingly accepted that the pathogenesis of glaucoma is multifactorial because of the fact that glaucomatous optic neuropathy occurs at all the levels of IOP and that patients progress regardless of interventions to regulate IOP. The exact mechanism of glaucomatous damage due to platelet hyperaggregation is not clear. It has been speculated that microcirculatory defects, such as vasoconstriction and retention of blood, damage endothelial cells, leading to subendothelial collagen exposure, which triggers platelet aggregation, subsequently causing the ischemic injury of the optic nerve (5, 20). Here is another question. What may cause the hyperresponsiveness of platelets? A possible cause of platelet hyperreactivity in patients with glaucoma may involve the pigment epithelium-derived factor (PEDF), which is a multifunctional secreted protein that mediates neuroprotection and inhibition of angiogenesis in the retina (21). PEDF is significantly reduced in glaucomatous eyes (22), which may lead to increased platelet aggregability and the establishment of a thrombogenic state.

Last but not least, platelet aggregates may promote an elevated IOP by blocking the physiological pores of Schlemm's canal. Platelet is fundamental in the function of the inner wall of Schlemm's canal (23). Disruption or alteration in platelets' function may result in extensive pore occlusion and decreased aqueous humor drainage (24).

The strengths of this study include the following points: (1) the first study for systematic evaluation on the relationship between platelet parameters and the structural, functional changes in POAG; (2) the first study to demonstrate directly that platelet parameters' close relationships with the thickness of the peripapillary retinal nerve fiber layer and macular ganglion cell complex, using OCT as a clinically useful and reliable tool; and (3) the strict inclusion criteria for this study and the detailed and comprehensive clinical examination performed by glaucoma specialists for all the enrolled subjects. Our results are expected to bring new inspiration for the study of biomarkers and novel targets in glaucoma.

Although this study was the first study to investigate the relationship between platelet parameters and the structural, functional changes in POAG, we acknowledge that this study had some limitations. First, this was a single-center, retrospective study involving a relatively small sample size due to the strict inclusion criteria. Further studies with larger sample sizes and multicenter settings are required to further confirm our findings. Second, due to the inherent deficiencies of retrospective methods, we cannot judge the association between platelet parameters and disease progression. This question is worth further exploration based on the prospective approach. Third, antiglaucoma therapy has been shown to prevent or delay the development of retinal ganglion cell damage. Although all the subjects received antiglaucoma therapy in this study, this study did not collect detailed information on medicine usage for data analysis. Further study will be suggested to focus on the mechanism of platelet activation in glaucomatous optic neuropathy through animal models or pathological experiments.

In conclusion, this study found significantly negative association between PDW, MPV levels and RNFL, GCC thickness, as well as positive association between PDW, MPV levels, and cup/disk area ratio in patients with POAG. Our results suggested that platelet activation may contribute to glaucomatous optic neuropathy.

## Data Availability Statement

The original contributions presented in the study are included in the article/supplementary material, further inquiries can be directed to the corresponding author/s.

## Ethics Statement

The studies involving human participants were reviewed and approved by the Ethics Review Committee of Eye and Ear, Nose, Throat Hospital (EENT), Fudan University. The patients/participants provided their written informed consent to participate in this study.

## Author Contributions

YM and SL performed the statistical analysis, drafted this article, and interpreted the data. MS contributed to data collection, literature search, and statistical analysis. XS critically revised this article. SL and WC were involved in the design of this study and supervised this project. All authors contributed to the article and approved the submitted version.

## Funding

This work was supported by the Excellent Physician—Excellent Clinical Researcher Plan (SYA202004), the Shanghai Municipal Commission of Health and Family Planning (201840050), the State Key Program of National Natural Science Foundation of China (81430007), the subject of major projects of National Natural Science Foundation of China (81790641), the Shanghai Committee of Science and Technology of China (17410712500), and Shanghai Science and Technology Committee Foundation Grant (19411964600).

## Conflict of Interest

The authors declare that the research was conducted in the absence of any commercial or financial relationships that could be construed as a potential conflict of interest.

## Publisher's Note

All claims expressed in this article are solely those of the authors and do not necessarily represent those of their affiliated organizations, or those of the publisher, the editors and the reviewers. Any product that may be evaluated in this article, or claim that may be made by its manufacturer, is not guaranteed or endorsed by the publisher.
